# Microbe-enzyme synergistic fermentation enhances tobacco stem cell wall degradation by modulating enzymatic activity and microbial community structure

**DOI:** 10.3389/fbioe.2026.1732779

**Published:** 2026-01-27

**Authors:** Zongcan Yang, Bo Fu, Chunlai Wu, Wenzhao Liu, Sensen Zhao, Tingting Zhang, Yongming Xu, Yingjie Feng, Yunjie Wu, Yunge Jing, Huanhuan Wang

**Affiliations:** 1 Technology Center, China Tobacco Henan Industrial Co., Ltd., Zhengzhou, Henan, China; 2 College of Tobacco, Henan Agricultural University, Zhengzhou, Henan, China

**Keywords:** bacterial diversity, cell Wall Degradation, enzymatic activity, enzyme synergy, tobacco stem

## Abstract

The valorization of tobacco stems, a major agricultural by-product with high lignin content, is hindered by the recalcitrance of plant cell walls. Conventional approaches using single microbes or enzymes suffer from inefficiency, instability, and poor performance on lignin-rich substrates. To address this, we proposed a “microbe-enzyme synergistic” strategy by combining *Bacillus megaterium* (a multifunctional degrader) with its own extracellular enzymes. Three treatments were designed: microbe-only (T_b_), enzyme-only (T_e_), and microbe-enzyme combination (T_b&e_), with sterile water as the control (CK). Cell wall component contents, enzyme activities, and microbial community structure were analyzed after 7 days of fermentation. The T_b&e_ treatment achieved the most substantial degradation: 64.93% for cellulose, 57.89% for lignin, and 37.20% for pectin, significantly outperforming T_b_ and T_e_. It also exhibited the highest activities of cellulase, laccase, and pectinase. High-throughput sequencing revealed that T_b&e_ specifically enriched *Bacillus megaterium* (94.97% of the community) while suppressing non-functional competitors. Our findings demonstrate that the synergy creates a positive feedback loop: initial enzymatic hydrolysis facilitates microbial colonization, which in turn boosts sustained enzyme production. This study elucidates a mechanistic model for efficient biodegradation and provides an innovative strategy for the high-value utilization of high-lignin agricultural wastes.

## 1 Introduction

The plant cell wall, a complex network of lignin, cellulose, and pectin, presents a major barrier to efficient biodegradation. Lignin forms a recalcitrant, cross-linked aromatic matrix that embeds and protects polysaccharides like cellulose and pectin (Liu et al., 2022; Wang et al., 2023; Zhong et al., 2022), whose linear, ordered structures further resist degradation (Cosgrove, 2024; Kumar and Turner, 2014). Conventional strategies using single microbial strains or enzyme preparations often face significant limitations for degrading such complex biomass. Single microbes, while capable of targeting specific components (Dong et al., 2025; Lu et al., 2019; Ibrahim et al., 2014), frequently exhibit a prolonged lag phase and insufficient enzyme yield. Conversely, the application of exogenous enzymes alone is costly, lacks persistence, and struggles to penetrate intact cell wall structures. These challenges are particularly acute for lignin-rich feedstocks like tobacco stems, where the dense aromatic matrix severely hinders microbial and enzymatic access.

The microbe-enzyme synergistic approach integrates the complementary advantages of both agents to overcome these bottlenecks. This strategy combines the immediate hydrolytic action of enzymes with the adaptive, sustained enzyme-producing capacity of microbes, potentially enhancing degradation efficiency (Lin et al., 2020; Shan et al., 2021). While effective in systems like silage (Zhao et al., 2018), its application to high-lignin tobacco stems remains underexplored.

*Bacillus megaterium* represents a strategic candidate for such synergy, as it possesses a broad-spectrum enzymatic arsenal capable of degrading cellulose, pectin, and lignin (Xu et al., 2024; Tang et al., 2020). This study innovatively employs a **“self-synergy”** system, combining *B. megaterium* with its own extracellular enzymes. This design serves a dual purpose: (i) it utilizes a single, multifunctional strain to attack multiple cell wall components, simplifying the system compared to multi-strain consortia; and (ii) it establishes a controlled model to isolate the core synergy principle—**immediate enzymatic hydrolysis coupled with sustained microbial enzyme production**—free from the compatibility issues of heterologous enzymes.

We hypothesized that this self-synergy would significantly enhance the degradation of tobacco stem cell walls. To test this, we designed three treatments: *B. megaterium* alone (T_b_), its enzymes alone (T_e_), and their combination (T_b&e_). The **specific objectives** were to: (i) quantitatively evaluate and compare the degradation efficiency of cellulose, lignin, and pectin; (ii) monitor the dynamics of relevant enzyme activities (cellulase, laccase, pectinase); and (iii) analyze the associated shifts in microbial community structure and functional potential to elucidate the synergistic mechanism.

## 2 Materials and methods

### 2.1 Culture methods for test strains

The test strains were obtained from Henan Agricultural University and preserved in the China Center for Type Culture Collection (CCTCC No: M 2023458). **Preparation of seed liquid**: A single colony from a *Bacillus megaterium* culture plate was inoculated into Nutrient Agar (NA) liquid medium and incubated with shaking at 30 °C and 180 rpm for 12 h to obtain the first-stage seed culture. Using the same conditions, the first-stage seed culture was inoculated into 50 mL fresh NA liquid medium at a 2% (v/v) inoculum and shaken for 8 h to prepare the second-stage seed culture **Preparation of fermentation broth**: The second-stage seed culture of *Bacillus megaterium* was inoculated into 50 mL fermentation medium at a 2% (v/v) inoculum and cultured at 30 °C with shaking at 180 rpm for 24 h to obtain fermentation broth. **Preparation of crude enzyme solution and bacterial suspension**: The *Bacillus megaterium* fermentation broth was centrifuged at 8,000 *g* and 4 °C; the supernatant (non-sterile) was collected as the crude enzyme solution. An equal volume of distilled water was added to the remaining bacterial pellet, mixed thoroughly, and used as the bacterial suspension, and immediately tobacco stem fermentation operation.

### 2.2 Experimental design and treatment groups

To investigate the microbe-enzyme synergy, four distinct treatment groups were established as follows: the control group (CK), tobacco stems treated with an equivalent volume of sterile distilled water. The Enzyme-only group (T_e_), tobacco stems treated with the crude extracellular enzyme solution derived from *Bacillus megaterium* fermentation. The microbe-only group (T_b_), tobacco stems treated with the washed cell suspension of *Bacillus megaterium. The microbe-enzyme combination (T*_*b&e*_ *),* tobacco stems treated with a combined solution containing both the crude enzyme solution and the bacterial suspension.

### 2.3 Tobacco stem fermentation

The ratio of tobacco stems to treatment solution was 0.25 mL/g. All samples were fermented in an incubator at 30 °C and 65% humidity for 7 days. At the end of fermentation, samples were immediately frozen in liquid nitrogen and stored at −80 °C for subsequent analysis.

### 2.4 Determination of cell wall material content in tobacco stems

#### 2.4.1 Pectin was extracted from samples

A weighed sample was placed in a beaker, and ethanol absolute was added. The mixture was heated in a water bath at 85 °C for 10 min, cooled, and centrifuged at high speed; the supernatant was discarded. 67% (v/v) ethanol was added to the precipitate, and the mixture was incubated in a water bath at 85 °C for another 10 min. Centrifugation and supernatant discard were repeated until soluble carbohydrates were completely removed. The precipitate was transferred to a round-bottomed flask using 0.5 mol/L H_2_SO_4_ solution (pH 0.5), heated under reflux in a water bath at 85 °C for 1 h, and cooled. The pectin extract was transferred to a volumetric flask, and 0.5 mol/L H_2_SO_4_ solution (pH 0.5) was added to reach the marked volume 25 mL. The extract was centrifuged at high speed and filtered; the filtrate was retained. A 1 mL aliquot of the filtrate was diluted to 50 mL with distilled water. From this diluted solution, 1 mL was pipetted into a 25 mL glass test tube, followed by 0.25 mL of 1.0 g/L ethanol-carbazole chromogenic reagent and 5.0 mL of 0.5 mol/L H_2_SO_4_ solution. The test tube was vortexed for 2 min, incubated in a constant-temperature water bath at 85 °C for 20 min, and then rapidly cooled. The absorbance of the solution was measured at 525 nm.

#### 2.4.2 Cellulose was extracted from samples

Pulverized samples were placed in test tubes, and a mixed solution of 2.5 mL acetic acid (HAc) and 2.5 mL nitric acid (HNO_3_) was added. The samples were heated in a boiling water bath for 25 min, cooled, centrifuged, and the supernatant was discarded. The precipitate was rinsed with distilled water three times. To the precipitate, 10 mL of 10% (w/w) H_2_SO_4_ solution and 10 mL of 0.1 mol L^-1^ K_2_Cr_2_O_7_ solution were added. The mixture was heated in a boiling water bath for 10 min, and the test tubes were rinsed three times with distilled water. After cooling, 5 mL of 20% (w/w) KI solution and 1 mL of 0.5% (w/w) starch solution were added. The mixture was titrated with 0.2 mol L^-1^ Na_2_S_2_O_3_ solution. A blank control was prepared by titrating 10 mL of 0.1 mol L^-1^ K_2_Cr_2_O_7_ (pre-mixed with 10 mL of 10% (w/w) H_2_SO_4_ solution) (Jin et al., 2017).

The content of cellulose was calculated using the following equation:

$$\text{Cellulose}=K\times \left(a‐b\right)/\left(n\times 24\right)$$

K = the concentration of Na_2_S_2_O_3_ solution (mol/L), a = the volume of Na_2_S_2_O_3_ solution was titrated into blank sample (mL), b = the volume of Na_2_S_2_O_3_ solution was titrated into sample (mL), n = the mass of samples (g).

#### 2.4.3 Lignin was extracted from samples

10 mL of 1% (w/w) acetic acid (CH_3_COOH) was added to the samples, which were then shaken and centrifuged. The precipitate was washed with 5 mL of 1% (w/w) CH_3_COOH, and a 1:1 (v/v) mixed solution of ethanol and diethyl ether was added. After soaking, the supernatant was discarded. After drying the precipitate, 72% (w/w) H_2_SO_4_ was added, and the mixture was incubated at room temperature until all cellulose was dissolved. Distilled water (10 mL) was added, and the mixture was heated in a boiling water bath for 5 min. After cooling, 5 mL distilled water and 0.5 mL 10% (w/w) BaCl_2_ solution were added, followed by centrifugation (8,000 r/min, 10 min). 10 mL 10% (w/w) H_2_SO_4_ and 10 mL 0.1 mol/L K_2_Cr_2_O_7_ solution were added to the precipitate, which was then heated in a boiling water bath for 15 min. After cooling, the residual fraction was washed with 20 mL distilled water. 5 mL 20% (w/w) KI solution and 1 mL 0.5% (w/w) starch solution were added, and the mixture was titrated with Na_2_S_2_O_3_ solution. A blank control was prepared by titrating a mixture of 10 mL 10% (w/w) H_2_SO_4_ and 10 mL 0.1 mol/L K_2_Cr_2_O_7_ solution.

The content of lignin was calculated using the following equation:

$$\text{Lignin}=K\times \left(a‐b\right)/\left(n\times 24\right)$$

K = the concentration of Na_2_S_2_O_3_ solution (mol/L), a = the volume of Na_2_S_2_O_3_ solution was titrated into blank sample (mL), b = the volume of Na_2_S_2_O_3_ solution was titrated into sample (mL), n = the mass of samples (g).

### 2.5 Enzyme activity measurement methods

#### 2.5.1 Cellulase and pectinase activities assays

For cellulase activity determination, each test tube contained 300 μL of 1% (w/v) sodium carboxymethyl cellulose solution and 100 μL of enzyme solution. For pectinase activity determination, the substrate was replaced with 300 μL of 1% (w/v) pectin solution, while the enzyme solution volume (100 μL) remained unchanged. Both mixtures were incubated at 50 °C for 30 min to initiate the reaction, the buffer used was 0.1 moL/L citric acid-sodium citrate buffer (0.1 moL/L citric acid and 0.1 moL/L sodium citrate were mixed at a ratio of 8.2:11.8), and the reaction pH was 5.0. After reaction, 400 μL of 3,5-dinitrosalicylic acid (DNS) solution was added to each tube; the mixtures were heated in a boiling water bath at 100 °C for 5 min and cooled with running water. The solutions were diluted to a final volume of 10 mL, and the absorbance was measured at 540 nm to determine cellulase and pectinase activities, respectively, and inactivated enzyme solution as a blank control.

To construct standard curves for cellulase and pectinase activity quantification: A 1 mg mL^-1^ glucose standard solution (for cellulase calibration) and a 1 mg mL^-1^ D-galacturonic acid standard solution (for pectinase calibration) was prepared. Serial dilutions of each standard solution were performed. For each diluted standard, 400 μL of DNS solution was added; the tubes were heated in a boiling water bath for 5 min, immediately cooled with running water, and diluted to 5 mL with distilled water. The absorbance of all diluted standard solutions was measured at 540 nm using a microplate reader (Zeni et al., 2011).

#### 2.5.2 Laccase activities assays

Test tubes for laccase activity measurement contained 2.7 mL of 50 mmol/L sodium acetate -acetic acid buffer solution (pH 5.0), 200 μL of 1.0 mmol/L 2,2′-azino-bis (3-ethylbenzothiazoline-6-sulfonic acid) (ABTS) solution and 400 μL of enzyme solution. After a 2 min reaction, the absorbance of the resulting color was measured at 420 nm to determine laccase activity (Srivastava et al., 2013). One unit of laccase activity was calculated using the following equation:

$$\text{Laccaes activity}=\left(\Delta_{t}\times V_{1}\times 10^{6}\right)/\left(\varepsilon \times V_{2}\times N\times m\times t\right)$$

Where Δ*A* = change in absorbance over time *t*, *V*_1_ = volume of the reaction system (mL), *V*_2_ = volume of enzyme solution added (mL), *ε* = molar absorptivity at 420 nm (3.6 × 10^4^ M^-1^ cm^-1^), *N* = dilution factor of the enzyme solution, m = volume of buffer used for enzyme extraction (mL g^-1^), *t* = reaction time (min).

### 2.6 The scanning electron microscopy images

The screened strains were streaked to NA solid medium and cultured at 30 °C for 24 h to observe the colony morphology. The strain was inoculated into liquid medium, 30 °C, 180 rpm shaking culture for 24 h, and the bacteria were collected to observe the characteristics of the bacteria under electron microscope.

### 2.7 Library construction

The total genomic DNA from microbial communities was extracted using the E. Z.N.A.® Soil DNA Kit (Omega Bio-tek, Norcross, GA, U.S.) according to the manufacturer’s instructions from the tobacco stem of each treatment. The extracted genomic DNA was checked by 2% agarose gel electrophoresis, and its concentration and purity were measured using a NanoDrop 2000. The V3-V4 region of the 16S rRNA gene was amplified by PCR using the primers 338F (5′-ACT​CCT​ACG​GGA​GGC​AGC​AG-3′) and 806R (5′-GGACTACHVGGGTWTCTAAT-3′) (Yang et al., 2023). The PCR products were quantified using the QuantiFluor™-ST blue fluorescence quantitative system (Promega Corporation). The products were then mixed in appropriate proportions according to the sequencing requirements of each sample. The amplified sequence library was constructed using the NEXTFLEX Rapid DNA-Seq Kit (New England Biolabs Inc., Ipswich, MA, U.S.), and high-throughput sequencing was performed on the Illumina MiSeq PE300 sequencing platform (Illumina Corporation, San Diego, U.S.).

### 2.8 High-throughput data analysis

The raw paired-end sequencing reads were quality-controlled using fastp software. FLASH software was employed to merge the raw sequences, and UPARSE software (version 7.0) was used to perform OTU (Operational Taxonomic Unit) clustering at a 97% similarity level while removing chimeric sequences (Chen et al., 2018; Mago and Salzberg, 2011; Edgar, 2013). OTU taxonomic classification was annotated using the RDP 11.5 classifier with the 16S rRNA gene database, using a confidence threshold of 70%. The community composition of each sample was analyzed at different taxonomic levels (Wang et al., 2007). Functional prediction of the 16S sequences was performed using PICRUSt2 software (Caicedo et al., 2020). OTUs clustered at 97% similarity and annotated at a 70% confidence threshold primarily support reliable taxonomic assignment at the genus level; species-level annotations are provided for reference only and should not be interpreted as definitive. All subsequent community composition analyses and functional inferences in this study are based on genus-level or higher taxonomic units to ensure result reliability.

### 2.9 Statistical analysis

Three replicates were set in all experiments. Microbial diversity analysis was conducted using the online platform of “Majorbio” Bioinformatics Cloud. Alpha diversity indices such as Chao 1 and Shannon index were calculated using mothur software, and differences in alpha diversity between groups were analyzed using the Wilcoxon rank-sum test (Schloss et al., 2009). Beta diversity distance matrices were calculated using Qiime software, and PCoA plots were generated using the ggplot2 package (Version 2.2.1) in R (version 3.3.1) (Estaki et al., 2020). LEfSe analysis (Linear Discriminant Analysis Effect Size) was applied to identify significantly different bacterial taxa at levels from phylum to genus between groups (LDA>2, p *≤* 0.05). Correlation network analysis was performed based on species selected with Spearman correlation coefficients |r| > 0.6 and *p* ≤ 0.05 (Segata et al., 2011).

## 3 Results

### 3.1 Effects of different treatment groups on enzyme activities and cell wall component contents

**FIGURE 1:**
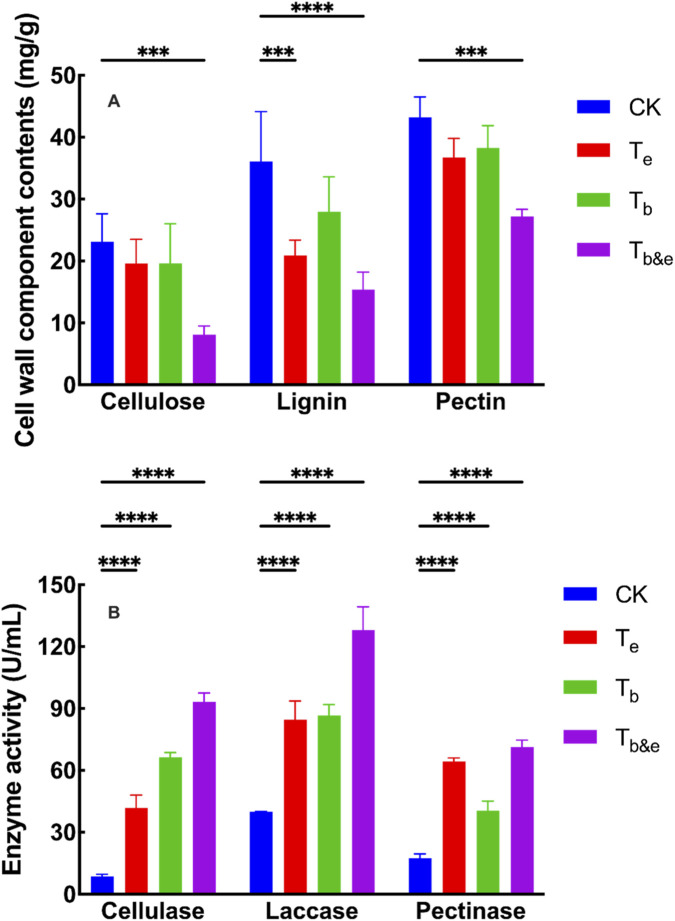
**Cell wall component contents and enzyme activities in tobacco stems after different fermentation treatments. (A)** Contents of cellulose, lignin, and pectin. **(B)** Activities of cellulase, laccase, and pectinase. CK: control; T_e_: enzyme-only; T_b_: microbe-only; T_b&e:_ microbe-enzyme combination. Data are presented as mean ± SD (n = 3). One-way ANOVA followed by Dunnett’s *post hoc* test was performed to compare all treatments with the CK group. ***p < 0.01.

To assess the content of cell wall materials in post-fermentation tobacco stems, the contents of cellulose, lignin, and pectin were determined for each treatment (Figure 1A). Compared with the CK group: Cellulose content decreased by 64.93% in the T_b&e_ group, 15.15% in the T_b_ group, and 6.19% in the T_e_ group; Lignin content decreased by 57.89% in the T_b&e_ group, 41.82% in the Te group, and 19.94% in the T_b_ group; Pectin content decreased by 37.20% in the T_b&e_ group, 11.63% in the T_e_ group, and 15.08% in the T_b_ group.

To investigate enzymatic activity in tobacco stems after different treatments, the activities of cellulase, laccase, and pectinase were measured after 7 days of fermentation (Figure 1B). Results showed that the activities of cellulase, pectinase, and laccase in the T_e_, T_b_, and T_b&e_ groups were all higher than those in the CK group. Notably, the T_b&e_ group (microbe-enzyme combination) exhibited higher cellulase, pectinase, and laccase activities than the T_e_ (enzyme-only) and T_b_ (microbe-only) groups.

In summary, the T_b&e_ T_b_ group (microbe-enzyme combination, with *Bacillus megaterium* as the functional microbe) had lower cellulose, lignin, and pectin contents than the T_b_ (microbe-only) and T_e_ (enzyme-only) groups. This result indicates that *Bacillus megaterium* plays a key role in the microbe-enzyme synergy system, and its combination with extracellular enzymes is well-adapted to tobacco stem cell wall degradation under the experimental conditions.

### 3.2 Effects of different treatment groups on microbial diversity

The Shannon and Simpson indices were used to describe community diversity across the four treatment groups (CK, T_e_, T_b_, T_b&e_), while Ace and Chao indices were used to evaluate community richness (Table 1). The Shannon index of T_b&e_ group was lower than other groups (P < 0.05), which was attributed to the addition of excessive enzyme suspension. Comparison of the Ace indices among the T_b_, T_e_, and T_b&e_ groups revealed that the community richness of the CK group was lower than that of the three experimental groups (*P* < 0.05), indicating a change in microbial abundance following treatment.

**TABLE 1:** Alpha diversity indices of microbial communities in tobacco stems under different treatments (mean ± standard deviation, n = 3).

Sample	ACE	Chao	Shannon	Simpson
CK	443.24 ± 183.95^a^	442.84 ± 184.88^a^	4.20 ± 0.44^a^	0.04 ± 0.02^c^
T_e_	342.51 ± 52.07^b^	341.68 ± 48.14^b^	1.81 ± 0.81^bc^	0.44 ± 0.30^b^
T_b_	344.64 ± 224.16^b^	345.54 ± 225.57^b^	2.78 ± 0.91^b^	0.25 ± 0.15^b^
T_b&e_	332.81 ± 116.91^b^	329.94 ± 117.47^b^	1.26 ± 0.99^c^	0.66 ± 0.28^a^

Different lowercase letters (a, b, c) indicate significant differences between groups (*P* < 0.05).

**FIGURE 2:**
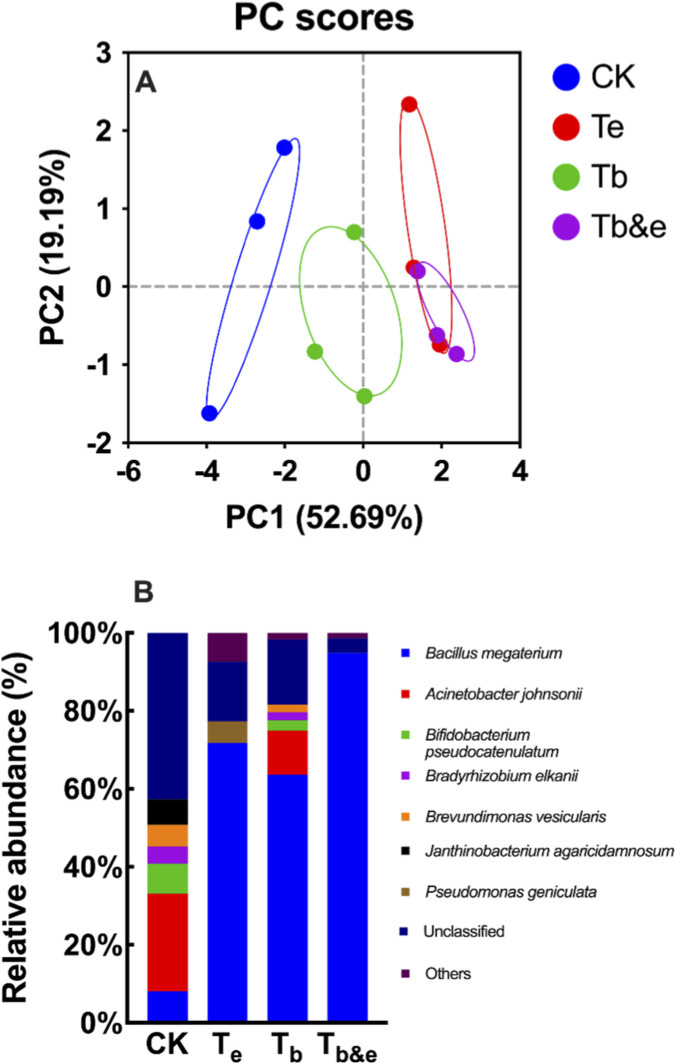
**Bacterial beta diversity of tobacco stems after fermentation. (A)** Principal Coordinate Analysis (PCoA) plot based on species-level community composition; **(B)** Community bar plot showing the relative abundance of dominant bacterial genera. CK: blank control; T_e_: enzyme-only treatment; T_b_: microbe-only treatment; T_b&e_: microbe-enzyme combination treatment.

To evaluate differences in the microbial community structure of tobacco stems among different treatment groups, PCoA was performed at the species level (Figure 2A). Axis 1 accounted for 52.69% of the total variation, and Axis 2 contributed 19.19%, indicating that these two axes effectively reflected differences in microbial community composition between treatment groups.

Figure 2B shows that the genus *Bacillus* (with the dominant OTU annotated as *Bacillus megaterium*, serving as a reference only) exhibited the highest relative abundance in the T_b&e_ group (94.97%), followed by the T_e_ group (71.88%), T_b_ group (63.61%), and CK group (8.13%). As the functional bacterium used in this study, the high abundance of the genus *Bacillus* in the T_b&e_ group is likely the key reason for the effective cell wall degradation in this group. In contrast, the genus *Acinetobacter* (with the dominant OTU annotated as *Acinetobacter johnsonii*) showed the highest relative abundance in the T_b_ group (11.33%), while its abundance in the T_b&e_ group was <1%, suggesting that enzyme addition in the T_b&e_ group may have inhibited the growth of the genus *Acinetobacter* to some extent. However, the crude enzyme solution was not filter-sterilized and therefore contained residual viable *B. megaterium* cells, which proliferated during the 7-day fermentation, leading to the observed high abundance in the T_e_ group.

These results indicate that different treatment groups selectively regulate the abundance of specific microbial genera in tobacco stems, and the T_b&e_ group can specifically enrich functional microbes (represented by *Bacillus megaterium*), which may serve as the core microbial driver for efficient degradation of cell wall components in tobacco stems under T_b&e_ treatment group.

### 3.3 Microbial function prediction

**FIGURE 3:**
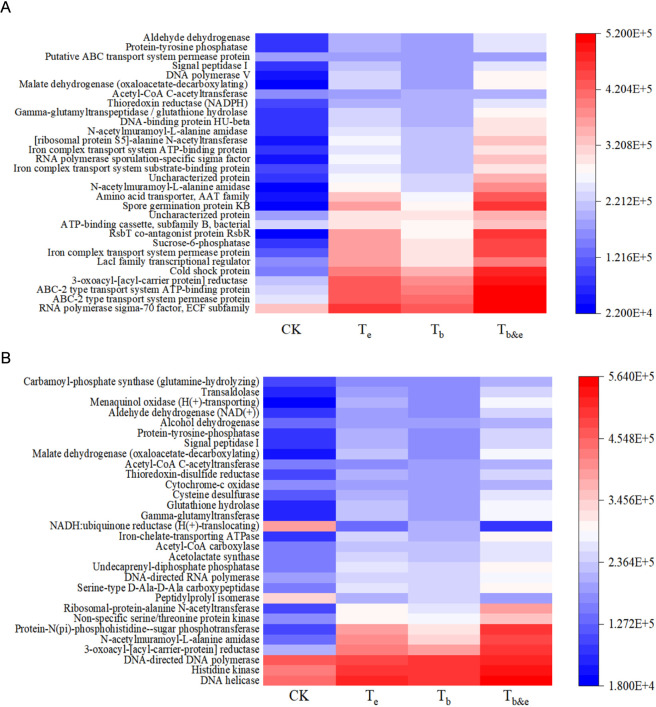
Functional prediction analysis of tobacco stem-associated microbial communities. **(A)** KO (KEGG Orthology) functional enrichment analysis; **(B)** Enzyme functional enrichment analysis. CK: blank control; T_e_: enzyme-only treatment; T_b_: microbe-only treatment; Tb&e: microbe-enzyme combination treatment.

To predict the functions of tobacco stem-associated microbial communities, PICRUSt2 software was used to analyze potential microbial functions based on the relationship between functional information and microbial abundance (Figure 3). Compared with the CK group, the microbial communities in the treatment groups showed a significant predicted enrichment of functional modules based on PICRUSt2 analysis (Figure 3A). Among these, cell wall hydrolysis and carbon metabolism are strongly associated with the predicted cell wall hydrolysis potential.

Figure 3B shows that the microbial communities in the treatment groups had a significant predicted enrichment of functional potential related to enzymes capable of cleaving carbon-nitrogen bonds based on PICRUSt2, with the highest predicted enrichment observed in the T_b&e_ group. Additionally, the treatment groups showed a significant predicted enrichment of functional potential related to enzymes involved in transmembrane transport, again with the highest predicted enrichment in the T_b&e_ group. These results indicate that the microbial functions in the treatment groups are significantly enriched in cell wall degradation-related functional modules, which facilitates the degradation of tobacco stem cell walls.

### 3.4 Correlation analysis of microbial abundance and cell wall substance content

**FIGURE 4:**
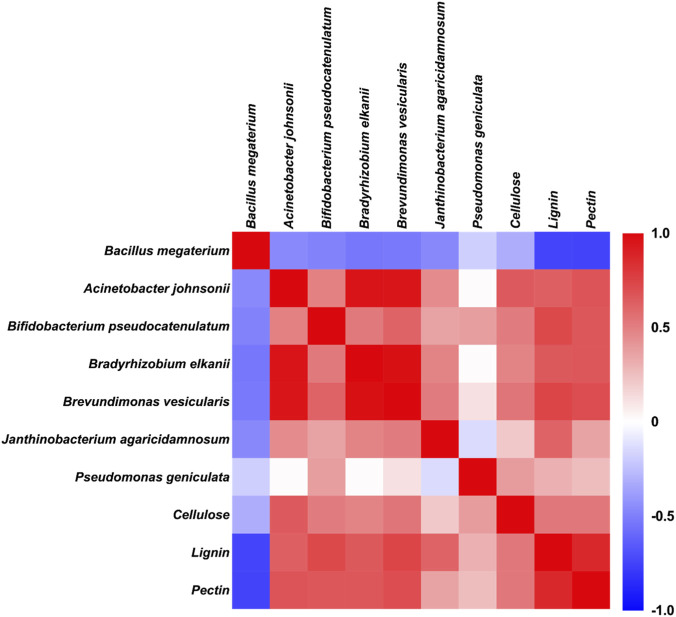
Nonparametric Spearman correlation analysis between microbial abundance and cell wall substance content. Color intensity indicates the strength of the correlation; positive correlations are shown in red, and negative correlations in blue.

To further clarify the interaction between microbial communities and cell wall component degradation, Spearman correlation analysis was performed between dominant microbial genera and the contents of cellulose, lignin, and pectin (Figure 4).

The genus *Bacillus* (core functional taxon in the T_b&e_ group, with the dominant OTU annotated as *Bacillus* megaterium) exhibited a negative correlation with cellulose and lignin contents. This reinforces the role of the genus *Bacillus* in degrading complex cell wall polysaccharides, as increased the genus *Bacillus* abundance coincided with degraded cellulose and lignin levels. In contrast, the genus *Acinetobacter* (dominant taxon in the T_b_ group, with the dominant OTU annotated as *A. johnsonii*) showed positive correlations with cellulose, lignin, and pectin contents, indicating its limited contribution to cell wall degradation and potential competitive inhibition by *Bacillus megaterium* in the T_b&e_ group.

Other genera, such as *Bradyrhizobium* (with reference annotation *Bradyrhizobium elkanii*) and *Brevundimonas* (with reference annotation *Brevundimonas vesicularis*), displayed positive correlations with multiple cell wall components, suggesting they may thrive in environments with abundant undegraded substrates but lack specialized degradation capabilities. The genus *Pseudomonas* (with reference annotation *Pseudomonas geniculata*) showed a positive correlation with pectin content.

These correlations confirm that *Bacillus megaterium* acts as the key driver of cell wall degradation in the microbe-enzyme synergy system, while other microbes exhibit either competitive or non-functional relationships with cell wall components.

## 4 Discussion

**FIGURE 5:**
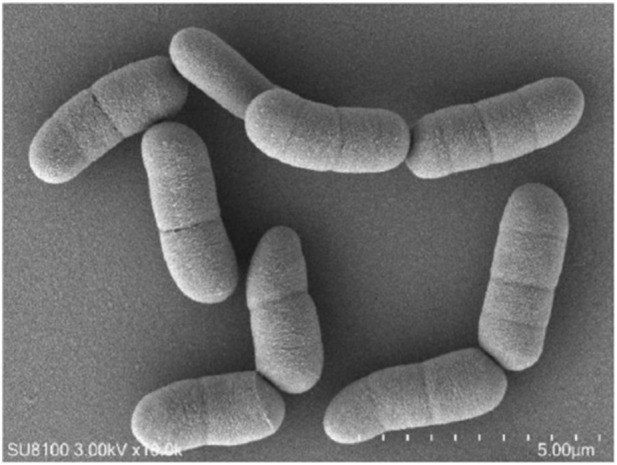
The scanning electron microscopy images.

This study aimed to resolve the inefficient degradation of high-lignin tobacco stems by designing a targeted microbe-enzyme synergistic system. Our results unequivocally demonstrate that the **combined application of** *Bacillus megaterium* (Figure 5) **and its enzymes (T**_**b&e**_ **)** outperformed either component alone, achieving superior cell wall deconstruction and establishing a functionally dominant microbial community. This validates our core hypothesis and provides a concrete solution to the limitations of single-agent (T_e_, T_b_) approaches outlined in the introduction.

Quantitatively, the T_b&e_ group achieved reductions of 64.93% in cellulose, 57.89% in lignin, and 37.20% in pectin relative to the CK group (Figure 1A). These degradation rates were 2.1–8.3-fold higher than those observed in the T_e_ and T_b_ groups. Concomitantly, the T_b&e_ group exhibited the highest activities of cellulase, laccase, and pectinase among all groups (Figure 1B), establishing a direct link between elevated cell wall-degrading enzyme activity and enhanced substrate decomposition efficiency. This observation aligns with the structural complexity of plant cell walls: lignin forms a highly cross-linked three-dimensional network of phenylpropanoid subunits, while cellulose and pectin exist as rigid linear polysaccharides linked by glycosidic bonds—features that collectively impede degradation (Cosgrove, 2024). The microbe-enzyme synergy in the T_b&e_ group overcomes these barriers by integrating the “immediate hydrolytic capacity” of exogenous enzymes and the “sustained enzyme synthesis” of *Bacillus megaterium*, creating a complementary metabolic loop.

Notably, the T_b_ group showed limited efficiency, likely due to **insufficient initial enzyme output** during the critical early phase (Xu et al., 2024; Zhang et al., 2024), whereas the Te group’s effect was transient, lacking the **self-replicating and adaptive capacity** of living microbes. Only the T_b&e_ group successfully integrated the **“immediate attack”** of enzymes with the **“sustained campaign”** of microbial growth and enzyme synthesis, leading to a persistent and enhanced degradation dynamic. These findings suggest that microbe-enzyme synergy resolves the intrinsic limitations of single-agent treatment: enzymes initiate the breakdown of recalcitrant cell wall structures (Zhao et al., 2024), while *Bacillus megaterium* utilizes the resulting small-molecule metabolites to proliferate and secrete additional enzymes—sustaining a positive feedback cycle that drives efficient and stable degradation.

The choice to employ a “self-synergy” system, utilizing *Bacillus* megaterium in conjunction with its own extracellular enzymes, warrants clarification in the context of lignocellulose degradation strategies. It is recognized that the complete decomposition of complex plant biomass in natural environments often involves synergistic microbial consortia. However, the primary aim of this study was to establish and validate a fundamental synergy mechanism using a simplified, well-defined system, rather than to immediately pursue maximum degradation efficiency via a fully optimized, multi-source enzyme combination. The selection of B. megaterium was strategic; this strain exhibits inherent multi-functionality with a documented capacity to degrade cellulose, pectin, and lignin. This makes it an ideal “minimal functional unit” for probing synergistic interactions without the additional complexity of cross-strain dynamics. The significant degradation achieved herein (Figure 1A) demonstrates that this single-strain-based synergy is substantially more effective than its individual components (microbe or enzyme alone), confirming the potency of the approach.

This “self-synergy” model presents distinct advantages: it inherently ensures enzyme-microbe compatibility, replicates the concept of a single, self-sufficient biocatalytic agent, and establishes a clear causal link between the introduced biological component and the observed functional outcome. It serves as a critical proof-of-concept and a mechanistic baseline. Future research can strategically build upon this foundation by designing heterologous synergy systems, where enzymes from specialized hyper-producers are combined with robust, adaptable microbes like B. megaterium for enhanced or targeted activity. This study confirms that a judiciously chosen single strain, operating within a synergistic framework with its own enzymes, can drive substantial biomass deconstruction. This offers a robust, simplified, and tractable alternative for applications where the management of multi-strain consortia or the cost of complex enzyme combinations presents practical challenges.

Beta diversity analysis via PCoA demonstrated that four treatment groups significantly altered the tobacco stem-associated microbial community structure (Figure 2A). From the sample distribution pattern in the PCoA plot, the CK group was clearly separated from the three treatment groups, suggesting that treatment groups significantly altered the microbial community structure of tobacco stems compared with the non-fermented control. Among the fermentation groups, the T_b_ group showed the most distinct separation from the T_e_ and T_b&e_ groups; in contrast, the T_e_ and T_b&e_ groups were relatively closely distributed, indicating that the microbial community structure of the T_b&e_ group differed more significantly from that of the single-enzyme or single-microbe treatment groups, and indicating that microbe-enzyme synergy reshapes the microbial community in a manner distinct from single-agent treatment.

At the species level, the T_b&e_ group was characterized by the highest relative abundance of *Bacillus megaterium*, whereas *A. johnsonii*—the dominant taxon in the T_b_ group—was significantly suppressed in the T_b&e_ group (Figure 2B). Correlation analysis further confirmed a significant negative correlation between *Bacillus megaterium* abundance and cellulose content (Figure 4), validating its role as the core driver of cell wall degradation. The suppression of the genus *Acinetobacter* (with reference annotation *A. johnsonii*) in the T_b&e_ group is likely attributed to niche differentiation induced by enzyme addition: exogenous enzymes accelerate the consumption of labile organic matter in tobacco stems, creating a nutrient environment that favors the growth of the genus *Bacillus* (with reference annotation *Bacillus megaterium*) over the genus *Acinetobacter* This selective enrichment of functional microbes highlights the ability of microbe-enzyme synergy to “engineer” microbial communities toward functional dominance. Future studies will adopt ASV analysis to enhance taxonomic resolution, particularly for exploring rare taxa or fine-scale species interactions. However, the current OTU-based analysis, combined with strict quality control and genus-level interpretation, is sufficient to address the core research question of this study—i.e., the link between microbe-enzyme synergy, community structure shifts, and cell wall degradation.

Functional prediction using PICRUSt2 further supported these mechanisms (Figures 3A,B). Compared with the CK group, all treatment groups showed a significant predicted enrichment of functional modules associated with cell wall hydrolysis (Encoding cell wall substance degradation enzyme-related metabolic pathways) and carbon metabolism based on this prediction method. The T_b&e_ group also exhibited the highest enrichment of enzymes involved in N-acetylmuramoyl-L-alanine amidase and Iron-chelate-transporting ATPase. **The significant enrichment of cellulose-hydrolyzing gene and carbon-nitrogen bond-cleaving enzyme in the T**_**b&e**_ **group confirms that the synergy upregulates these key metabolic pathways, which enhance the breakdown of recalcitrant cell wall structures and subsequent nutrient uptake by *Bacillus megaterium***. These enrichments indicate that microbe-enzyme synergy not only increases the abundance of functional microbes but also upregulates their metabolic pathways related to cell wall degradation. The elevated abundance of transmembrane transport enzymes suggests enhanced uptake of degraded cell wall fragments by *Bacillus megaterium*, further fueling its growth and enzyme production. This “microbe-enzyme-community-function” cascade explains why the T_b&e_ group achieves superior degradation efficiency compared to treatments that modify only enzyme activity or microbial abundance in isolation. The functional inferences in this study rely on PICRUSt2, which infers metabolic potential based on 16S rRNA gene community composition and existing genome databases, rather than directly detecting functional genes via metagenomics or enzyme activity via biochemical assays. Therefore, the ‘enrichment of functional modules or enzyme-related potential’ reported herein reflects a hypothetical functional trend of the community, which provides a preliminary clue for understanding the microbe-enzyme synergy mechanism but requires further validation by metagenomic sequencing or targeted enzyme activity assays in future studies.

The integrated data from this study allow us to propose a multi-stage mechanism for the enhanced degradation observed in the T_b&e_ group. This “self-synergy” operates through a positive feedback loop: Stage 1 (Priming): The co-applied extracellular enzyme combination provides immediate hydrolytic and oxidative attack. Laccase and other lignin-modifying enzymes begin to disrupt the dense lignin matrix, while cellulase and pectinase weaken the polysaccharide network. This initial “priming” breaks the physical barrier, increasing substrate accessibility. Stage 2 (Microbial Colonization and Amplification): The oligosaccharides and simpler compounds released from Stage 1 serve as readily available nutrients, promoting the rapid colonization and proliferation of the inoculated *Bacillus megaterium*, as evidenced by its dramatic enrichment in the T_b&e_ community (Figure 2B). Stage 3 (Sustained and Targeted Degradation): The established, abundant population of *B. megaterium* nitiates robust *in-situ* synthesis and secretion of a wide spectrum of cell wall-degrading enzymes (Figure 1B). The functional prediction of enriched hydrolytic and transmembrane transport modules (Figure 3) supports this shift towards a dedicated degradation metabolism. The microbe now acts as a localized, self-replicating enzyme factory, driving further deconstruction. Stage 4 (Community Shaping): This altered nutrient landscape and micro-environment (e.g., potential pH shifts, metabolic by-products) selectively favors functional degraders like *Bacillus*while suppressing non- or weak-degrading competitors like *Acinetobacter* (Figures 2B, 4), thereby engineering a microbial community optimized for the degradation task. This cascade—from enzymatic priming to microbial amplification to community reshaping—explains why the synergistic effect is greater than the sum of its parts and provides a mechanistic framework for applying such strategies to other recalcitrant biomasses.

The findings of this study are consistent with but extend previous research on microbe-enzyme synergistic fermentation (Du et al., 2025). Supplementing corn stover silage with lactic acid bacteria (*Lactobacillus plantarum*) and cellulase improves fermentation quality (Liu et al., 2024), a study that demonstrated the potential of synergy but focused on single cell wall components and specific crops. Notably, the lignin content of tobacco stems is higher than that of corn stover; the lignin reduction rate of the T_b&e_ group (57.89%) in this study was significantly higher than that of previous microbe-enzyme combination treatment on corn stover. This result suggests that the synergistic system with *Bacillus megaterium* as the core is well-adapted to tobacco stems (a high-lignin raw material), which is consistent with the strain’s functional characteristics of degrading multiple cell wall components. This study also systematically evaluated the degradation of three core cell wall components (cellulose, lignin, pectin), providing a comprehensive understanding of how synergy overcomes the structural complexity of plant cell walls—an advantage over previous plant stem degradation studies that relied primarily on single microbes or enzymes (Abdelrhim et al., 2024) and achieved maximum cellulose degradation rates lower than those of the T_b&e_ group. The novelty of this study stems from the selection of *Bacillus* megaterium, which degrades multiple macromolecules (cellulose, lignin, pectin) (Saha et al., 2010) and complements the broad-spectrum hydrolytic activity of its extracellular enzymes. Unlike single-function microbes, *Bacillus megaterium* eliminates the need for multi-strain consortia in the tobacco stem fermentation system, simplifying processes and improving stability. This characteristic makes it well-adapted to the designed microbe-enzyme synergy system and provides practical support for subsequent industrial application of tobacco stem degradation. While the fermentation time of maize cobs was shortened by microbe-enzyme synergy, the underlying dynamics of the microbial community or functional genes were not explored (Lin et al., 2020). This study fills this gap by demonstrating that synergy reshapes microbial communities toward functional dominance and enriches key metabolic pathways—providing a mechanistic basis for optimizing synergy in agricultural by-product treatment.

The novelty of this work is twofold. **First, at the conceptual level,** we designed and validated a **“self-synergy” system** using a single, multifunctional bacterium paired with its own enzymes. This model elegantly decouples and then reintegrates the two key processes—initial hydrolysis and sustained biocatalysis—offering a **novel mechanistic lens** to study microbe-enzyme interactions, distinct from systems employing heterogeneous microbial consortia or commercial enzymes. **Second, at the applicative level,** we successfully targeted **tobacco stems, a high-lignin feedstock** that has been understudied in this context. The remarkable 57.89% lignin reduction achieved underscores the strategy’s **unique efficacy against recalcitrant aromatics**, filling a significant knowledge and technology gap. These insights advance the fundamental understanding of synergistic biodegradation and provide a **translatable framework** for developing efficient, low-cost bioprocesses for lignocellulosic waste valorization.

While this study demonstrated the efficacy of the microbe-enzyme synergy through quantitative reduction of major cell wall components and associated biological analyses, certain methodological aspects warrant further consideration to deepen the insights. An important experimental consideration is that the tobacco stem substrate was not sterilized prior to fermentation. This design choice was made to better simulate a practical, non-aseptic bioprocessing scenario for agricultural waste. We acknowledge that this introduces the indigenous microbiome of the stems as an additional variable. However, several internal lines of evidence support the conclusion that the observed effects are primarily driven by the introduced treatments, particularly the synergy in the T_b&e_ group: (i) the inoculated *Bacillus megaterium*achieved overwhelming and reproducible dominance specifically in the T_b&e_ group (94.97% relative abundance, Figure 2B), indicating a strong selective advantage conferred by the treatment combination; (ii) the control (CK) group developed a distinct and stable community structure (Figures 2A,B), demonstrating that uncontrolled stochastic contamination did not homogenize the samples; and (iii) the strong negative correlation between *B. megaterium* abundance and substrate content (Figure 4) functionally links the success of this specific organism to the degradation outcome. Nevertheless, to unequivocally attribute all community dynamics and degradation metrics solely to the introduced agents, future foundational studies should employ sterilized substrates under axenic or gnotobiotic conditions.

Furthermore, complementary analytical techniques could provide even deeper insights in future work. Fourier-transform infrared (FTIR) spectroscopy could be employed to directly monitor the breakdown of key chemical bonds (e.g., lignin aromatic rings, cellulose C-O-C linkages) within the solid residue, offering spectroscopic validation of structural deconstruction. Quantitative analysis of released hydrolysis products, such as reducing sugars and phenolic monomers, would not only provide direct evidence of enzymatic cleavage but also allow for the mapping of carbon flow and metabolic pathways during the synergistic process. Incorporating these approaches in subsequent studies will yield a more comprehensive, mechanistic understanding of the degradation process.

## 5 Conclusion

The microbe-enzyme synergistic fermentation technology developed in this study provides an eco-friendly and cost-effective solution for plant stem valorization: reducing cell wall component content enhances the suitability of treated stems for high-value applications, such as ruminant feed (Soest, 1985; Jung and Allen, 1995), biofuel or biological feedstocks (Gomez et al., 2008; Acker and Vanholme, 2013), or organic fertilizers. Beyond tobacco stems, the core principle of this study—using microbe-enzyme synergy to engineer functional microbial communities for enhanced cell wall degradation—can be adapted to other lignocellulosic by-products (Zhong et al., 2022). Collectively, this study systematically demonstrates that *Bacillus megaterium*-enzyme synergistic fermentation plays a key role in tobacco stem cell wall degradation, with the strain showing good adaptability to the synergy system. It also clarifies the underlying mechanisms (enhanced enzyme activity, reshaped microbial community, and elevated functional potential), and provides a theoretical and technical basis for the high-value utilization of low-value agricultural by-products.

It is important to note the scope of this study. Our primary objective was to evaluate the **macro-level efficacy and microbial ecological dynamics** of the proposed synergy. Consequently, we focused on quantifying the **disappearance of primary substrates** (cellulose, lignin, pectin) rather than profiling the full suite of **hydrolysis products**. The fate of these degradation products is inferred to be assimilation by the microbial community, fueling the observed growth and functional shifts. Future research employing **metabolomics** or **targeted chemical analysis** will be invaluable to map the complete degradation pathways and quantify carbon flow. Additionally, while PICRUSt2 provides strong predictive insights, **metagenomic or metatranscriptomic** analyses could directly verify the expression of the functional genes implicated in the synergistic process.

## Data Availability

The datasets presented in this study can be found in online repositories. The names of the repository/repositories and accession number(s) can be found below: https://www.ncbi.nlm.nih.gov/, PRJNA1209677.

## References

[B1] Abdelrhim A. S. Hemeda N. F. Ali Mwaheb M. Omar M. O. A. Dawood M. F. A. (2024). The role of Trichoderma koningii and Trichoderma harzianum in mitigating the combined stresses motivated by sclerotiniasclerotiorum and salinity in common bean (phaseolusvulgaris). Plant Stress 11, 100370. 10.1016/j.stress.2024.100370

[B2] Acker R. V. Vanholme R. Storme V. Mortimer J. C. Dupree P. Boerjan W. (2013). Lignin biosynthesis perturbations affect secondary cell wall composition and saccharification yield in Arabidopsis thaliana. Biotechnol. Biofuels 6 (1), 1–17. 10.1186/1754-6834-6-46 23622268 PMC3661393

[B3] Caicedo H. H. Hashimoto D. A. Caicedo J. C. Pentland A. Pisano G. P. (2020). PICRUSt2 for prediction of metagenome functions. Nat. Biotechnol. 38 (6), 685. 10.1038/s41587-020-0548-6 32483366 PMC7365738

[B4] Chen S. Zhou Y. Chen Y. Gu J. (2018). Fastp: an ultra-fast all-in-one FASTQ preprocessor. Bioinformatics 34 (17), i884–i890. 10.1093/bioinformatics/bty560 30423086 PMC6129281

[B5] Cosgrove D. J. (2024). Structure and growth of plant cell walls. Nat. Rev. Mol. Cell Biol. 25 (5), 19–358. 10.1038/s41580-023-00691-y 38102449

[B24] Demment M. W. Soest D. P. J. V. (1985). A nutritional explanation for body-size patterns of ruminant and nonruminant herbivores. Am. Nat. 125 (5), 641–672. 10.1086/284369

[B6] Dong L. Jing Y. Hou J. Zhou J. Yu T. Chen S. (2025). A dominant subgroup of marine Bathyarchaeia assimilates organic and inorganic carbon into unconventional membrane lipids. Nat. Microbiol. 10, 2579–2590. 10.1038/s41564-025-02121-5 40973791

[B7] Du Z. Cui S. Chen Y. Zhang Y. Wang S. Yan X. (2025). Fermentation regulation: revealing bacterial community structure, symbiotic networks to function and pathogenic risk in corn stover silage. Agriculture 15 (16), 1791. 10.3390/agriculture15161791

[B8] Edgar R. C. (2013). UPARSE: highly accurate OTU sequences from microbial amplicon reads. Nat. Methods 10 (10), 996–998. 10.1038/nmeth.2604 23955772

[B9] Estaki M. Jiang L. Bokulich N. A. Mcdonald D. Knight R. Kosciolek T. (2020). QIIME 2 enables comprehensive end‐to-end analysis of diverse microbiome data and comparative studies with publicly available data. Curr. Protoc. Bioinforma. 70 (1), e100. 10.1002/cpbi.100 32343490 PMC9285460

[B10] Gomez L. D. Steele‐King C. G. McQueen‐Mason S. J. (2008). Sustainable liquid biofuels from biomass: the writing’s on the walls. New Phytol. 178. 10.1111/j.1469-8137.2008.02422.x 18373653

[B11] Ibrahim D. Weloosamy H. Sheh-Hong L. (2014). Potential use of nylon scouring pad cubes attachment method for pectinase production by Aspergillus niger HFD5A-1. Process Biochem. 49 (4), 660–667. 10.1016/j.procbio.2014.01.012

[B12] Jin X. Chen X. Shi C. Li M. Guan Y. Yu C. Y. (2017). Determination of hemicellulose, cellulose and lignin content using visible and near infrared spectroscopy in Miscanthus sinensis. Bioresour. Technol. 241 (October), 603–609. 10.1016/j.biortech.2017.05.047 28601778

[B13] Jung H. G. Allen M. S. (1995). Characteristics of plant cell walls affecting intake and digestibility of forages by ruminants. J. Animal Sci. 73 (9), 2774–2790. 10.2527/1995.7392774x 8582870

[B14] Kumar M. Turner S. (2014). Plant cellulose synthesis: CESA proteins crossing kingdoms. Phytochemistry 112 (1), 91–99. 10.1016/j.phytochem.2014.07.009 25104231

[B15] Lin B. Yan J. Zhong Z. Zheng X. (2020). A study on the preparation of microbial and nonstarch polysaccharide enzyme synergistic fermented maize cob feed and its feeding efficiency in finishing pigs. BioMed Res. Int. 2020 (2), 1–11. 10.1155/2020/8839148 33274228 PMC7683112

[B16] Liu K. Zhuang Y. Chen J. Yang G. Dai L. (2022). Research progress on the preparation and high-value utilization of lignin nanoparticles. Int. J. Mol. Sci. 23 (13), 7254. 10.3390/ijms23137254 35806259 PMC9266533

[B17] Liu X. D. Wang A. F. Zhu L. Q. Guo W. Guo X. J. Zhu B. (2024). Effect of additive cellulase on fermentation quality of whole-plant corn silage ensiling by a bacillus inoculant and dynamic microbial community analysis. Front. Microbiol. 10.3389/fmicb.2023.1330538 38264477 PMC10803609

[B18] Lu J. Yang Z. Xu W. Shi X. Guo R. (2019). Enrichment of thermophilic and mesophilic microbial consortia for efficient degradation of corn stalk. J. Environ. Sci. 78 (April), 118–126. 10.1016/j.jes.2018.07.010 30665630

[B19] Mago T. Salzberg S. L. (2011). FLASH: fast length adjustment of short reads to improve genome assemblies. Bioinformatics 27 (21), 2957–2963. 10.1093/bioinformatics/btr507 21903629 PMC3198573

[B20] Saha S. Roy R. N. Sen S. K. Ray A. K. (2010). Characterization of cellulase-producing bacteria from the digestive tract of tilapia, Oreochromis mossambica (peters) and grass carp, Ctenopharyngodon idella (valenciennes). Aquac. Res. 37 (4), 380–388. 10.1111/j.1365-2109.2006.01442.x

[B21] Schloss P. D. Westcott S. L. Ryabin T. Hall J. R. Hartmann M. Hollister E. B. (2009). Introducing mothur: open-source, platform-independent, community-supported software for describing and comparing microbial communities. Appl. and Environ. Microbiol. 75, 7537–7541. 10.1128/AEM.01541-09 19801464 PMC2786419

[B22] Segata N. Izard J. Waldron L. Gevers D. Miropolsky L. Garrett W. S. (2011). Metagenomic biomarker discovery and explanation. Genome Biol. 12 (6), R60. 10.1186/gb-2011-12-6-r60 21702898 PMC3218848

[B23] Shan S. Yang L. Zhang X. Shi J. Li H. Li Z. (2021). Inhibitory effect of bound polyphenol from foxtail millet bran on miR-149 methylation increases the chemosensitivity of human colorectal cancerHCT-8/Fu cells. Mol. Cell. Biochem. 476 (2), 513–523. 10.1007/s11010-020-03906-4 33011952

[B25] Srivastava P. Andersen P. C. Marois J. J. Wright D. L. Srivastava M. Harmon P. F. (2013). Effect of phenolic compounds on growth and ligninolytic enzyme production in botryosphaeria isolates. Crop Prot. 43 (January), 146–156. 10.1016/j.cropro.2012.09.015

[B26] Tang A. Haruna A. O. Majid N. M.Ab. Jalloh M. B. (2020). Potential PGPR properties of cellulolytic, nitrogen-fixing, phosphate-solubilizing bacteria in rehabilitated tropical forest soil. Microorganisms 8 (3), 442. 10.3390/microorganisms8030442 32245141 PMC7143980

[B27] Wang Q. George G. Tiedje J. Cole J. R. (2007). Naive bayesian classifier for rapid assignment of rRNA sequences into the new bacterial taxonomy. Appl. Environ. Microbiol. 10.1128/AEM.00062-07 PMC1950982 17586664

[B28] Wang Z. Tang H. Liu G. Gong H. Li Y. Chen Y. (2023). Compound probiotics producing cellulase could replace cellulase preparations during solid-state fermentation of millet bran. Bioresour. Technol. 385, 129457. 10.1016/j.biortech.2023.129457 37422095

[B29] Xu P. Shu L. Yang Y. Kumar S. Tripathi P. Mishra S. (2024). Microbial agents obtained from tomato straw composting effectively promote tomato straw compost maturation and improve compost quality. Ecotoxicol. Environ. Saf. 270, 115884. 10.1016/j.ecoenv.2023.115884 38154152

[B79] Yang L. Yang G. Wang J. Xiong B. Guo P. Wang T. (2023). Seasonal changes in total mercury and methylmercury in subtropical decomposing litter correspond to the abundances of nitrogen-fixing and methylmercury-degrading bacteria. J. Hazard. Mater. 442, 130064. 10.1016/j.jhazmat.2022.130064 36182885

[B30] Zeni J. Cence K. Elis Grando C. Tiggermann L. Colet R. Lerin L. A. (2011). Screening of pectinase-producing microorganisms with polygalacturonase activity. Appl. Biochem. Biotechnol. 163 (3), 383–392. 10.1007/s12010-010-9046-5 20669053

[B31] Zhang Y. Kang J. Chen H. X. (2024). Wheat-origin bacillus community drives the formation of characteristic metabolic profile in high-temperature daqu. J. Bangladesh Coll. Physicians Surg. 191 (January), 1–10. 10.1016/j.lwt.2023.115597

[B32] Zhao J. Dong Z. Li J. Chen L. Bai Y. Jia Y. (2018). Ensiling as pretreatment of rice straw: the effect of hemicellulase and Lactobacillus plantarum on hemicellulose degradation and cellulose conversion. Bioresour. Technol. 266 (October), 158–165. 10.1016/j.biortech.2018.06.058 29966925

[B33] Zhao S. Zhang T. Hasunuma T. Kondo A. Zhao X. Q. Feng J. X. (2024). Every road leads to rome: diverse biosynthetic regulation of plant cell wall-degrading enzymes in filamentous fungi Penicillium oxalicum and Trichoderma reesei. Crit. Rev. Biotechnol. (5/8), 44. 10.1080/07388551.2023.2280810 38035670

[B34] Zhong Y. Wang T. Yan M. Miao C. Zhou X. Tong G. (2022). High-value utilization of bamboo pulp black liquor lignin: preparation of silicon-carbide derived materials and its application. Int. J. Biol. Macromol. 217 (September), 66–76. 10.1016/j.ijbiomac.2022.07.045 35835306

